# Managed Long-Term Services and Supports and Caregiving Among Dually Enrolled Older Adults

**DOI:** 10.1001/jamanetworkopen.2025.28006

**Published:** 2025-08-21

**Authors:** Andrew D. Jopson, Chanee D. Fabius, Jennifer L. Wolff

**Affiliations:** 1 Department of Health Policy and Management, Johns Hopkins Bloomberg School of Public Health, Baltimore, Maryland

## Abstract

**Importance:**

Managed Long-Term Services and Supports (MLTSS) has been implemented by more than half of all state Medicaid programs, but knowledge of its scope and consequences for caregiving is limited.

**Objective:**

To describe observed trends in MLTSS program presence and care hours among older adults dually enrolled in Medicare and Medicaid between 2012 and 2022.

**Design, Setting, and Participants:**

This repeated cross-sectional study used data from the 2012-2022 National Health and Aging Trends Study (NHATS) linked with county-level measures of MLTSS program presence. Included in the study were adults aged 70 years or older dually enrolled in Medicare and Medicaid (hereafter older dual enrollees) who were living in the community and receiving assistance with self-care or mobility activities. Data analyses were conducted from December 2023 to June 2025.

**Exposure:**

Residence in an area with MLTSS program presence.

**Main Outcomes and Measures:**

The primary outcome was the total care hours per week received by older dual enrollees estimated as the mean (SD) care hours received per week overall and by caregiver type for each survey round. Percentages were weighted to account for the complex survey design.

**Results:**

Among 2549 participants, the mean (SD) age was 80.3 (8.5) years, 70.1% were female, and 26.5% lived alone. The weighted percentage of community-living, older dual enrollees receiving assistance who resided in areas with an MLTSS program increased from 39.4% in 2012 to 71.4% in 2022. Older dual enrollees receiving assistance were helped by a mean (SD) of 2.8 (1.6) caregivers and received 65.6 care hours per week (weighted percentage of total care hours, 80.9%). Fifty percent of participants received paid help. Most care hours were provided by unpaid family caregivers (mean [SD] care hours per week, 42.0 [74.0]). Mean (SD) weekly care hours for those residing in areas with continuous MLTSS presence were higher than those living in areas that added or had no MLTSS program presence (72.9 [82.8] vs 61.6 [71.7] and 60.0 [85.0] hours, respectively; *P* = .03). Among those residing in areas with continuous MLTSS, mean (SD) care hours provided by paid family caregivers increased steadily over the observation period from 2.0 (11.0) hours in 2012 to 23.8 (57.9) hours in 2022.

**Conclusions and Relevance:**

This cross-sectional study found increases in the percentage of older dual enrollees receiving assistance in the community and residing in areas with MLTSS program presence, with persistent heavy reliance on unpaid family caregivers. Systems are needed to monitor the care experiences of patients and their caregivers.

## Key Points

**Question:**

Did Managed Long-Term Services and Supports (MLTSS) program presence and care hours change for older adults dually enrolled in Medicare and Medicaid who were receiving assistance in the community between 2012 and 2022?

**Findings:**

This cross-sectional study of 2549 participants in the National Health and Aging Trends Study found that the share of older dual Medicare and Medicaid enrollees receiving assistance in the community and residing in areas with MLTSS program presence increased from 39.4% in 2012 to 71.4% in 2022. Unpaid family caregivers were the predominant source of care hours in all years.

**Meaning:**

The increasing share of older dual Medicare and Medicaid enrollees residing in areas with MLTSS program presence underscores the need for systems to monitor their experiences and those of family caregivers who provide the majority of care hours.

## Teaser

This cross-sectional study uses data from the National Health and Aging Trends Study to describe trends in the presence of the Managed Long-Term Services and Supports program and care hours among older adults dually enrolled in Medicare and Medicaid.

## Introduction

Approximately 7.7 million adults aged 65 and older who are dually enrolled in Medicare and Medicaid (hereafter older dual enrollees) account for disproportionate Medicaid spending on long-term services and supports (LTSS), primarily due to the high cost of nursing home care.^1^ For this reason, state Medicaid programs have pursued payment models to encourage LTSS delivery at home and reduce reliance on institutional settings.^2^ Over the past 2 decades, managed LTSS (MLTSS) has been embraced by states to control spending growth, expand access to home and community-based services (HCBS), and improve care coordination and efficiency.^3,4,5,6,7,8^

Under MLTSS programs, managed care organizations receive a fixed payment to deliver and coordinate LTSS for eligible beneficiaries. Although MLTSS programs may vary in structure, services, and populations served, they seek to bridge gaps in a fragmented LTSS delivery system by providing beneficiaries with a care coordinator who can assess care needs, develop a person- and family-centered plan, and coordinate services.^6,7,8^ The approach to implementing an MLTSS program is decided by states, which may limit the geographic reach to specific counties or decide whether program enrollment is mandatory or voluntary for eligible beneficiaries.^9^Information about the structure and scope of MLTSS programs has been compiled, but does not shed light on the care experiences of those residing in areas in which a MLTSS program has been adopted: information that is critical to understanding programmatic reach, characteristics of the population and their care needs, and program effects.^5,9,10^

To date, data constraints have hindered understanding of the characteristics of older dual enrollees residing in areas with MLTSS programs.^11^ Publicly available enrollment numbers do not distinguish dual enrollees aged 65 or older in their reports, making it difficult to ascertain how many older dual enrollees are or could be served by MLTSS programs.^12^ Additionally, amid increasing state adoption of these programs, it is critically important to understand how caregiving receipt (ie, receipt of paid help, number of caregivers, weekly care hours received) among older dual enrollees living in the community has changed in relation to MLTSS program presence. This analysis seeks to overcome this evidence gap by drawing on data from a national survey to estimate trends in caregiving among older dual enrollees receiving assistance in the community from 2012 to 2022 by residence in areas with MLTSS program presence.

## Methods

This cross-sectional study received ethics approval from the Human Subjects Division at the Johns Hopkins Bloomberg School of Public Health with a waiver of informed consent because it involved no more than minimal risk to participants and could not otherwise be reasonably carried out. This study followed the Strengthening the Reporting of Observational Studies in Epidemiology (STROBE) reporting guidelines.

### Data and Study Population

We used data from the National Health and Aging Trends Study (NHATS) rounds 2-12, corresponding to annual data collection in 2012 through 2022. NHATS is a longitudinal, nationally representative survey of Medicare beneficiaries aged 65 years or older that gathers information on health, functioning, and helpers. Our sample included participants who self-reported dual Medicare-Medicaid enrollment and received assistance with 1 or more self-care activities (eating, bathing, toileting, dressing) or mobility (transferring, getting around inside) while living in the community. We restricted our sample to participants aged 70 years or older in each survey year to account for an aging study population and enable comparable trends over the observation period.^13^ Analyses of caregiving measures were limited to those living in the community due to differences in care in residential care settings and nursing homes.^14^

### Measures

#### MLTSS Program Presence

To assess county-level measures of MLTSS program presence, we extracted information from the Centers for Medicare & Medicaid Services Medicaid Managed Care Enrollment and Program Characteristics Reports from 2013 to 2021 for 2 managed care program types that are available to full benefit, dual-eligible enrollees: comprehensive managed care organization and MLTSS or MLTSS only.^12^ These reports describe the geographic reach of managed care programs in each state, as well as whether program enrollment was mandatory or voluntary. Mandatory enrollment means that states mandate eligible beneficiaries to enroll in managed care (ie, MLTSS). Voluntary enrollment means eligible beneficiaries can opt in or opt out (passive enrollment). Our review found that 19 states had mandatory MLTSS programs and 3 states had voluntary MLTSS programs by 2021.

County-level indicators of MLTSS program presence (yes or no) and enrollment type (mandatory, voluntary, or no MLTSS) were assembled for each year and linked to NHATS participant place of residence by county Federal Information Processing Standard codes in the NHATS geographic files. As reports were not available for 2012 and 2022, we drew on MLTSS measures from the closest available years (2013 and 2021). Because multiple states adopted MLTSS programs during the observation period, we classified counties into 3 MLTSS subgroups: (1) continuous MLTSS, (2) added MLTSS, and (3) no MLTSS. Counties with continuous MLTSS had MLTSS programs present in both 2012 and 2022. Counties in the subgroup that added MLTSS programs did not have these programs in 2012 but did in 2022. Counties in the subgroup with no MLTSS programs did not have any MLTSS programs in 2012 or 2022.

#### Sociodemographic and Health Characteristics

We examined older adults’ sociodemographic characteristics (sex, age, race and ethnicity), living arrangement (alone vs with others), health (dementia status and self-reported chronic conditions), and functional status (number of self-care and mobility activities requiring assistance). Race and ethnicity were included in this study as part of the sociodemographic description of the cohort were self-reported as follows: Hispanic or other race and ethnicity, non-Hispanic Black, and non-Hispanic White. The Hispanic or other category included those of Alaskan Native, American Indian, Asian, Hispanic, Native Hawaiian, Pacific Islander, or other race and ethnicity. These race and ethnicity categories were combined to comply with data use agreement export requirements. Participants were classified as having dementia if they met 1 of 3 criteria: self-reported dementia or Alzheimer disease diagnosis, AD8 Dementia Screening interview score of 2 or higher (range: 0 to 8, with score of 2 or higher indicating probable dementia), or a series of cognitive tests administered at the survey interview, as previously described.^15^ NHATS gathers information about chronic conditions by asking whether participants have been told by their clinicians that they have heart disease, high blood pressure, had a heart attack, arthritis, osteoporosis, lung disease, cancer, diabetes, and stroke. Any “yes” response to heart disease, high blood pressure, or a heart attack was interpreted as an indication of any cardiac condition. We categorized the number of chronic conditions as 0 to 2 conditions, 3 to 4 conditions, or 5 or more conditions.

#### Caregiving Characteristics

The NHATS Other Person file collects information about individuals who assist with care activities, the weekly care hours they provide, their relationship to the older adult, and whether they were paid. For this study, we referred to all individuals providing help for health and functioning reasons as caregivers. We calculated the total number of unique caregivers who assisted an NHATS participant with self-care, mobility, household, medical care, or transportation. Receipt of paid help identifies older adults receiving assistance from at least 1 paid caregiver. We used these measures to calculate the number of paid and unpaid caregivers. We categorized caregivers as family (eg, spouse, children) or other (friends, neighbors, paid aides) based on their relationship to the NHATS participant. Finally, we differentiated 4 caregiving types based on relationship and payment: paid family, unpaid family, paid other, and unpaid other.

Our primary outcome of interest was total care hours per week, which was calculated by adding care hours received from all caregivers within a given month and dividing the total by 4.3 to derive weekly care hours. For participants whose caregivers had missing valid care hours (4.6%), we used an imputation strategy made available by NHATS.^16^

### Statistical Analysis

We constructed a pooled sample of participants from included survey rounds. We described sociodemographic and health characteristics of older dual enrollees receiving help in our pooled sample and then estimated the annual percentage of older dual enrollees residing in areas with an MLTSS program presence, including whether enrollment was mandatory or voluntary, stratified by survey round (eTables 1 and 2 in Supplement 1). We then described characteristics of the overall cohort and stratified by MLTSS program presence (participants’ residence in areas with continuous MLTSS, added MLTSS, or no MLTSS). We conducted Pearson χ^2^ tests for categorical measures and 1-way analysis of variance for continuous measures across the 3 groups. The threshold for statistically significant global differences was set at 2-sided *P* < .05. Finally, we estimated the mean and mean square error (MSE) care hours received per week overall and by caregiver type (unpaid family caregiver, paid family caregiver, unpaid other caregiver, and paid other caregiver) for each survey round (eTable 3 in Supplement 1). We examined annual estimates of care hours by residence in areas with continuous MLTSS, added MLTSS, or no MLTSS. Because our goal was to describe observed trends, we did not conduct tests of statistical significance in trend analyses. We used NHATS’ analytic weights, which account for the survey’s complex sampling design and nonresponse to estimate a nationally representative sample for each year. Statistical analyses were conducted from December 2023 to June 2025 using Stata/MP, version 18.1 (Stata Corp).

## Results

Among 2549 participants included in the analysis, the mean (SD) age was 80.3 (8.5); the survey-weighted percentages of participants were 70.1% of participants were female, 42.0% were Hispanic or other individuals, 21.2% were non-Hispanic Black individuals, 36.8% were non-Hispanic White individuals, and 26.5% lived alone (Table 1). In all, 34.0% participants had dementia, 80.4% had 3 or more chronic conditions, and the mean (SD) number of self-care or mobility activities that required assistance was 2.8 (2.0). Compared with older dual enrollees receiving assistance in the community who resided in areas without an MLTSS program, those who resided in areas that added or had continuous MLTSS programs were more likely to be of Hispanic or other race and ethnicity (53.9% of individuals residing in areas with continuous MLTSS and 56.1% residing in areas with added MLTSS vs 11.5% residing in areas with no MLTSS, *P* < .001).

**Table 1.** Characteristics of Study Cohort by Residence in an Area With MLTSS Program Presence

Characteristic	No. (weighted %)^a^				*P* value^c^
	Overall Cohort	No MLTSS^b^	Added MLTSS^b^	Continuous MLTSS^b^	
Observations, No.	2549	907	732	910	NA
Age, mean (SD), y	80.3 (8.5)	79.7 (9.4)	80.4 (8.0)	80.6 (8.2)	.47
Sex					
Male	626 (29.9)	194 (27.1)	207 (31.6)	225 (30.7)	.64
Female	1923 (70.1)	713 (72.9)	525 (68.4)	685 (69.3)	
Race and ethnicity^d^					
Hispanic or other^e^	736 (42.0)	68 (11.5)	329 (56.1)	339 (53.9)	<.001
Non-Hispanic Black	1162 (21.2)	564 (34.9)	249 (15.1)	349 (15.6)	
Non-Hispanic White	651 (36.8)	275 (53.6)	154 (28.8)	222 (30.4)	
Living arrangement					
Alone	646 (26.5)	256 (30.2)	196 (24.9)	194 (25.0)	.55
With others	1903 (73.5)	651 (69.8)	536 (75.1)	716 (75.0)	
Dementia status					
No dementia	1583 (66.0)	575 (70.7)	466 (65.4)	542 (63.0)	.17
Dementia	966 (34.0)	332 (29.3)	266 (34.6)	368 (37.0)	
Chronic conditions^f^					
0-2	562 (19.6)	200 (20.0)	135 (19.3)	227 (19.5)	.77
3-4	1460 (57.7)	519 (56.3)	437 (61.1)	504 (56.2)	
≥5	527 (22.7)	188 (23.8)	160 (19.5)	179 (24.2)	
No. of self-care or mobility activities receiving assistance, mean (SD)^g^	2.8 (2.0)	2.6 (2.1)	2.8 (2.0)	2.9 (2.0)	.06

Abbreviations: MLTSS, Medicaid managed long-term services and supports; NA, not applicable.

^a^ Percentages were weighted to account for the complex survey design.

^b^ Counties with continuous MLTSS had MLTSS programs present throughout the study period, while those with added MLTSS added MLTSS programs during the study period. Counties with no MLTSS did not have any MLTSS programs present during the study period.

^c^ *P* values represent global tests for differences across groups: survey-weighted χ^2^ tests for categorical variables and survey-weighted 1-way analysis of variance tests for continuous variables.

^d^ Percentages do not sum to 100% due to rounding.

^e^ Other category includes Asian, American Indian, Native Hawaiian, Pacific Islander, Multiracial, or other race or ethnicity specified by the participant.

^f^ NHATS gathers information about chronic conditions by asking participants, “Has a doctor ever told you that you have the following diseases:” heart disease, high blood pressure, had a heart attack, arthritis, osteoporosis, lung disease, cancer, diabetes, and stroke. Any “yes” response to heart disease, high blood pressure, or had a heart attack was categorized as indication of any cardiac condition.

^g^ Self-care activities included eating, bathing, toileting, and dressing. Mobility activities included bed transfer and walking inside the home.

**Figure 1.**
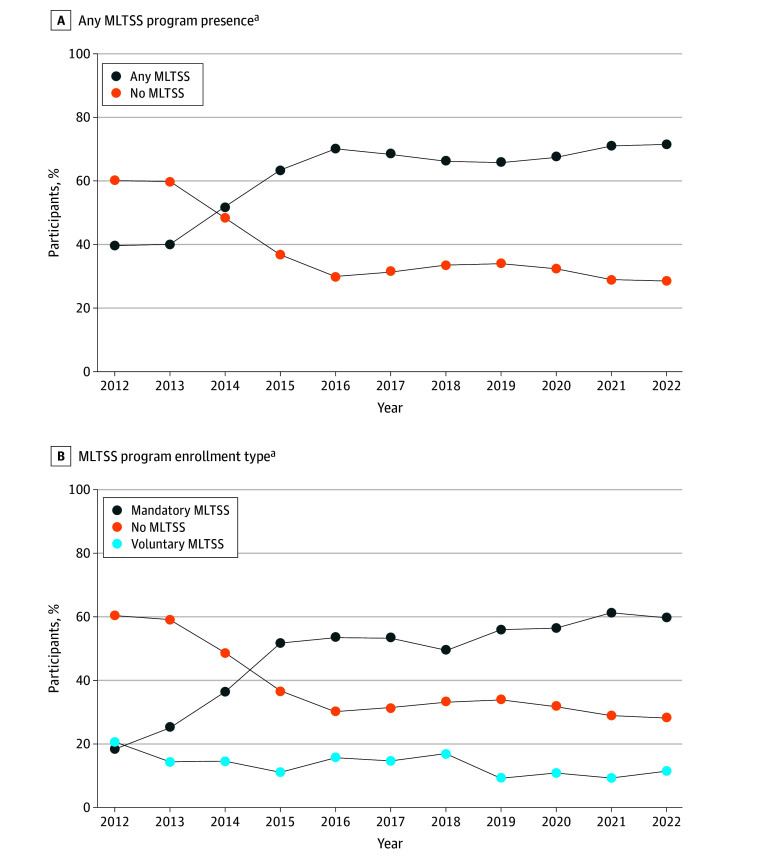
Percentage of Older Dual Enrollees Receiving Assistance in the Community By Residence in an Area With MLTSS Program Presence, 2012-2022

We estimate that the number of older dual enrollees receiving assistance in the community increased from 913 000 in 2012 to more than 1.1 million in 2022 (Figure 1A). The weighted share of older dual enrollees residing in areas with MLTSS program presence increased from 39.4% in 2012 to 71.4% in 2022, reflecting an increase from a weighted estimate of 360 000 enrollees in 2012 to 818 000 in 2022. The weighted percentage of older dual enrollees residing in areas with mandatory MLTSS enrollment increased from 18.8% in 2012 to 59.8% in 2022 (Figure 1B), while the weighted percentage of enrollees residing in areas with voluntary MLTSS enrollment decreased from 20.6% in 2012 to 11.6% in 2022.

Half of older dual enrollees received paid help (Table 2). Those who received assistance were helped by a mean (SD) of 2.8 (1.7) caregivers and received a mean (SD) of 65.6 (80.9) care hours per week. Unpaid family caregivers provided a mean (SD) of 42.0 (74.0) care hours per week. No statistically significant differences were observed in receipt of paid help or number of caregivers by MLTSS subgroups. However, mean (SD) weekly care hours for those residing in areas with continuous MLTSS were higher relative to those living in areas that added or had no MLTSS program presence (72.9 [82.8] vs 61.6 [71.7] and 60.0 [85.0] hours, respectively; *P* = .03). Group differences in mean (SD) care hours were predominantly due to lower use of paid family care among those who resided in areas without MLTSS vs areas that added or had continuous MLTSS (4.6 [27.9] hours vs 10.1 [33.1] hours and 9.1 [31.9] hours, respectively; *P* = .01).

**Table 2.** Caregiving Characteristics of Study Cohort by Residence in Area With MLTSS Program Presence

Caregiving characteristics	Overall Cohort	No MLTSS^a^	Added MLTSS^a^	Continuous MLTSS^a^	*P* value^b^
Observations, No.	2549	907	732	910	NA
Receipt of paid help, No. (weighted %)^c^					
No	1247 (50.0)	493 (54.2)	302 (47.4)	452 (48.9)	.49
Yes	1302 (50.0)	414 (45.8)	430 (52.6)	458 (51.1)	
Total No. of caregivers, mean (SD)^d^	2.8 (1.7)	2.9 (1.9)	2.8 (1.7)	2.7 (1.5)	.15
Paid caregivers	0.7 (1.0)	0.7 (1.1)	0.7 (0.9)	0.7 (0.9)	.95
Unpaid caregivers	2.2 (1.7)	2.3 (1.9)	2.2 (1.7)	2.1 (1.5)	.19
Care hours received per week, mean (SD)					
Total from all caregivers	65.6 (80.9)	60.0 (85.0)	61.6 (71.7)	72.9 (82.8)	.03
Paid family caregivers	8.1 (31.8)	4.6 (27.9)	10.1 (33.1)	9.1 (31.9)	.01
Unpaid family caregivers	42.0 (74.0)	41.1 (79.2)	40.0 (66.0)	44.1 (75.1)	.76
Paid other caregivers	10.5 (30.4)	9.3 (30.1)	7.7 (24.8)	13.5 (33.3)	.15
Unpaid other caregivers	5.1 (22.2)	4.9 (25.0)	3.8 (14.9)	6.1 (24.4)	.16

Abbreviations: MLTSS, Medicaid managed long-term services and supports; NA, not applicable.

^a^ Continuous MLTSS describes counties that had MLTSS programs present throughout the observation period. Added MLTSS describes counties that added MLTSS during the study period. No MLTSS describes counties that did not have any MLTSS programs present throughout the study period.

^b^ *P* values represent global tests for differences across groups: survey-weighted χ^2^ tests for categorical variables and survey-weighted 1-way analysis of variance tests for continuous variables.

^c^ Percentages were weighted to account for the complex survey design.

^d^ Caregivers were individuals who assisted with self-care, mobility, household, medical care, or transportation activities. Family caregivers included were spouses, children, or other extended family. Other caregivers included friends, neighbors, or paid care aides.

### Trends in Care Hours Received Per Week by Caregiver Type

**Figure 2.**
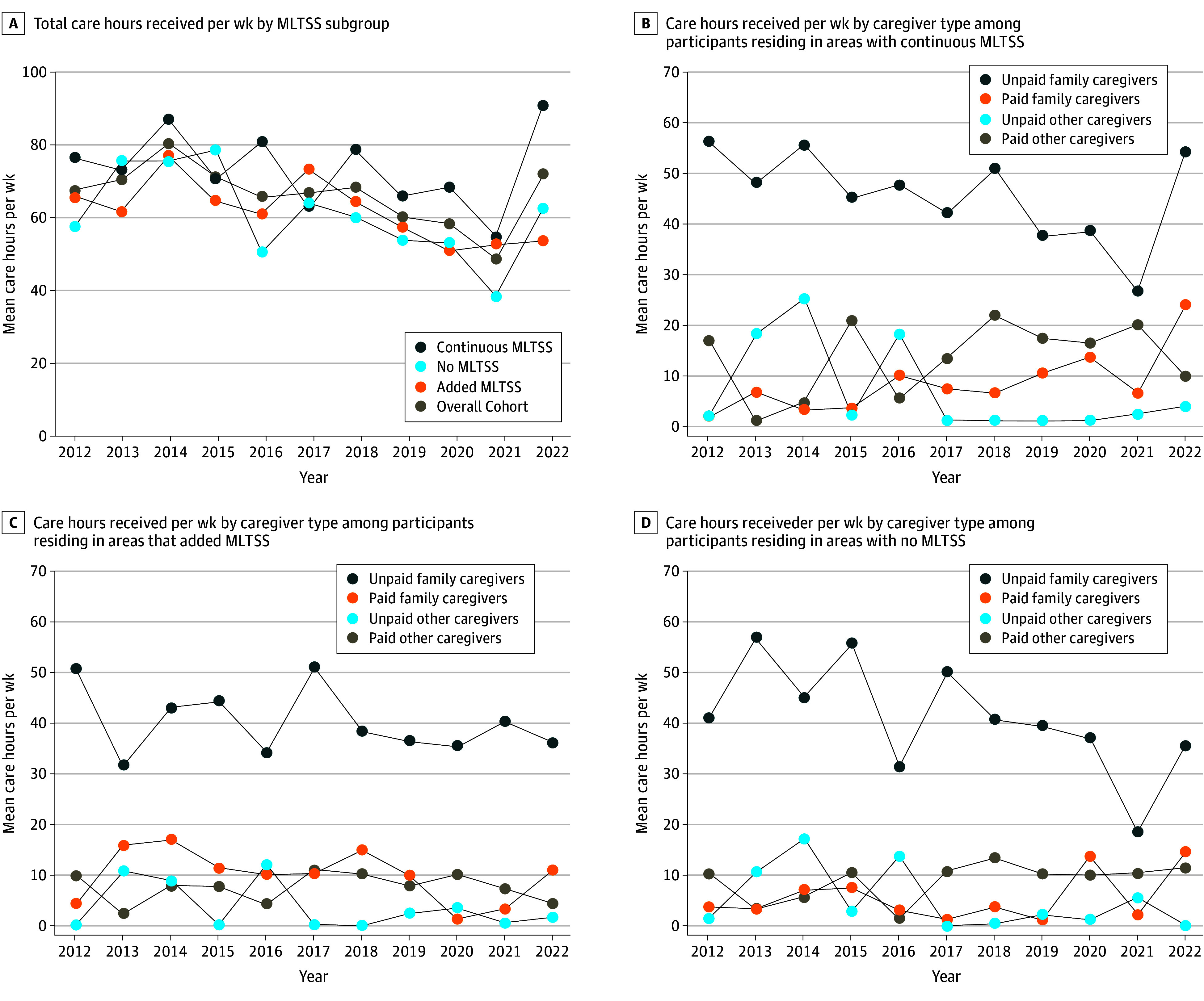
Care Hours Received Per Week Among Older Dual Enrollees Receiving Assistance in the Community

Among all older dual enrollees receiving assistance in the community, the mean (SD) total care hours received per week was high over the observation period regardless of MLTSS program presence (67.1 [93.0] hours in 2012 to 71.8 [99.4] hours in 2022) (Figure 2A). Unpaid family caregivers were the predominant providers of care hours in all years (Figure 2B-D). However, mean (SD) care hours from unpaid family caregivers decreased between 2012 and 2022 in each subgroup (continuous MLTSS, 55.4 [101.5] hours to 52.9 [103.4] hours; added MLTSS, 50.7 [77.5] hours to 36.2 [60.7] hours; no MLTSS, 41.1 [81.9] hours to 35.9 [63.0] hours). We observed dynamic patterns over time in mean care hours provided by paid family caregivers, paid other caregivers, and unpaid other caregivers. One exception was among dually enrolled older adults residing in areas with continuous MLTSS, for whom the mean (SD) care hours provided by paid family caregivers increased steadily from 2.0 (11.0) hours in 2012 to 23.8 (57.9) hours in 2022 (Figure 2B).

## Discussion

To our knowledge, this is the first national study to examine trends in MLTSS program presence and care hours received per week among older dual enrollees receiving assistance in the community. We found that the weighted percentage of older dual enrollees residing in areas with an MLTSS program nearly doubled from 39.4% in 2012 to 71.4% in 2022. This observed increase primarily occurred among those residing in areas with mandatory MLTSS enrollment (increasing from 18.8% in 2012 to 59.8% in 2022). Study findings reinforce the high care needs among older dual enrollees receiving assistance in the community who received more than 60 care hours per week from a mean of 2.8 caregivers. A key finding was that unpaid family caregivers were the predominant providers of care hours and that care hours provided by paid family and other paid caregivers increased over time, with such increases especially notable among those residing in areas with continuous MLTSS program presence. Altogether, our findings depict the increasing presence of MLTSS in the Medicaid LTSS landscape with family caregivers as the primary source of assistance for older dual enrollees receiving assistance in the community.

The persistent heavy reliance on unpaid family caregivers by older dual enrollees living in the community reinforces the importance of engaging and supporting families under MLTSS programs.^17^ One mechanism of support includes family caregiver assessments, which collect information about strengths, capabilities, needs, and challenges. Such assessments have not been adopted by all states^18,19^ despite being a foundational best practice to ensuring community living.^18,20,21^ Some states require that MLTSS programs conduct such assessments; however, there is variation in what information is gathered, and it is unclear how programs use this information to support caregivers’ training needs or well-being.^18,22^ Family caregiver assessments are typically conducted annually or when there is a substantial change in older adults’ care needs that may preclude timely interventions.^22^ In light of the demonstrated value of tailored assessment and support to family caregivers, more frequent monitoring of caregiving by MLTSS program coordinators could be valuable by prioritizing supports to caregivers with the greatest needs.^23,24^ Our finding that care hours provided by unpaid family caregivers decreased from 49.6 hours in 2012 to 43.4 hours in 2022 was surprising and should be considered with caution given our small sample size and some evidence that care hours provided by family have been stable over this period^25^ or even increased in the years following the COVID-19 pandemic.^26,27^

The increasing care hours provided by paid family caregivers to older dual enrollees residing in areas with continuous MLTSS is a finding that aligns with rebalancing initiatives.^28^ Growth in programs that enable family members to be paid for caregiving, such as through self-directed programs, aligns with the US Supreme Court’s *Olmstead v L.C.* (1999) decision and prioritization of HCBS in lieu of institutional care.^29^ While nearly all state Medicaid programs offered personal care benefits permitting payment of family members prior to the COVID-19 pandemic, pandemic-related policy changes expanded the availability and flexibility of these benefits.^30^ Improved care coordination through MLTSS programs may lead to greater awareness and use of HCBS, including personal care and self-directed service benefits that allow enrollees to hire and pay family members.^31,32^ Most MLTSS programs include some form of self-directed service benefits, but it is unclear to what extent older dual enrollees and their families are aware of or participate in these programs.^33,34^ The ability to hire and pay family caregivers is particularly important given the shortage of home care workers to meet increasing demand for HCBS^35^; however, understanding the allocation of constrained resources is an ongoing challenge for MLTSS and state Medicaid programs.

Our finding that the number of older dual enrollees receiving assistance in the community and residing in areas with MLTSS programs increased from 2012 to 2022 is a notable contribution to existing evidence about MLTSS. Our estimates differ from publicly available MLTSS enrollment data, which do not differentiate MLTSS enrollees by dual enrollment, age, or residential setting.^10^ The complexity of MLTSS programs by state leads to inconsistent reporting that makes it difficult to produce and assess national population estimates over time. While our study does not quantify actual MLTSS enrollment among this subpopulation, we offer insights about a subpopulation potentially impacted by increasing state adoption of MLTSS. Our finding that more than half of older dual enrollees receiving assistance in the community resided in areas with mandatory MLTSS enrollment by 2022 warrants special comment. While states may adopt mandatory MLTSS enrollment for financial and budgetary reasons,^36^ constrained choice may disrupt access to preferred and established health care professionals.^37^ Additionally, capitated payments under MLTSS programs may inhibit access to care, and concerns have been expressed that current oversight and monitoring are insufficient.^38,39^ On the other hand, mandatory MLTSS enrollment may expand care coordination, person-centered care planning, and improve connections to LTSS professionals and caregiver supports, thereby addressing care gaps that present challenges in a fee-for-service environment.^40^ More federal- and state-level monitoring and evaluation are needed to determine whether mandatory enrollment inhibits access to supports or preference-sensitive care.

### Limitations

This study has several limitations. First, survey data may be subject to recall bias. We relied on self-reported measures of Medicaid enrollment and functional status to identify our sample, which is subject to misclassification.^41^ We also did not consider state-level variation in LTSS benefits, such as state plans or HCBS waivers, that may be carved in or carved out from certain MLTSS programs. We recognize that the type of LTSS benefits offered within a state and individual preferences rather than the presence of MLTSS alone may affect caregiving receipt in the community. Available sample and NHATS data use guidelines limited the ability to make state-level estimates or evaluate state-based differences in MLTSS programs and relevant subgroups. We did not conduct any form of statistical testing of differences in trends over time. Despite these limitations, our study describes the dynamic MLTSS landscape using nationally representative survey data and how it relates to the characteristics and caregiving received by older dual enrollees.

## Conclusions

In this cross-sectional study of older dual enrollees receiving assistance in the community, care hours provided by unpaid family caregivers in the community were persistently high from 2012 to 2022. The increasing share of older dual enrollees residing in areas with MLTSS program presence underscores the need for systems to monitor the experiences of older dual enrollees and their family caregivers who provide the majority of care hours.

## Notes

Supplement 1. **eTable 1.** Percentage of Older Dual-Enrollees Receiving Assistance in the Community By Residence in Managed Long-term Services and Support (MLTSS) Program Presence and Survey Round **eTable 2.** Sociodemographic, Health, and Function Characteristics Among Older Dual-Enrollees Receiving Assistance in the Community by Survey Round **eTable 3.** Caregiving Characteristics Among Older Dual-Enrollees Receiving Assistance in the Community by Survey Round

Supplement 2.
Data Sharing Statement

## References

[1] Medicare Payment Advisory Commission (MedPAC) and Medicaid and CHIP Payment and Access Commission (MACPAC). Data book: beneficiaries dually eligible for Medicare and Medicaid. 2023. Accessed June 19, 2024. https://www.macpac.gov/wp-content/uploads/2023/02/Feb23_MedPAC_MACPAC_DualsDataBook-WEB-508.pdf

[2] Ryan J, Edwards BC. Rebalancing Medicaid long-term services and supports. *Health Affairs Forefront*. September 18, 2015. Accessed June 6, 2024. https://www.healthaffairs.org/do/10.1377/forefront.20150918.050656/full/

[3] Barth S, Silow-Carroll S, Reagan E, Russell M, Simmons T. Care coordination in integrated programs serving dually eligible beneficiaries–health pan standards, challenges and evolving approaches: report to the Medicaid and CHIP Payment and Access Commission. Health Management Associates. March 2019. Accessed September 9, 2022. https://www.macpac.gov/wp-content/uploads/2019/03/Care-Coordination-in-Integrated-Care-Programs-Serving-Dually-Eligible-Beneficiaries.pdf

[4] Archibald ND, Kruse AM, Somers SA. The emerging role of managed care in long-term services and supports. Public Policy Aging Rep. 2018;28(2):64-70. doi:10.1093/ppar/pry011

[5] Lewis E, Eiken S, Amos A, Saucier P. The growth of managed long-term services and supports programs: 2017 update. Medicaid.gov. January 29, 2018. Accessed January 24, 2021. https://www.medicaid.gov/medicaid/downloads/mltssp-inventory-update-2017.pdf

[6] Edwards BC, Sen AP. High demand and fragmentation: the current state of long-term services and supports in America. Generations. 2019;43(1):20-24.

[7] Dean KM, Grabowski DC. Care coordination for dually eligible beneficiaries. St Louis Univ J Health Law Policy. 2014;8(1):35-46.

[8] Musumeci M. Key themes in Medicaid managed long-term services and supports waivers. The Henry J. Kaiser Family Foundation. Accessed August 6, 2022. https://files.kff.org/attachment/key-themes-in-capitated-medicaid-managed-long-term-services-and-supports-waivers-issue-brief

[9] Chapter 3: Managed long-term services and supports: status of state adoption and areas of program evolution. In: *Report to Congress on Medicaid and CHIP*. MACPAC. June 2018. https://www.macpac.gov/wp-content/uploads/2018/06/June-2018-Report-to-Congress-on-Medicaid-and-CHIP.pdf

[10] Rosenstein D, Dickey J, Kirk S. Medicaid Managed Care enrollment and program characteristics, 2021. Mathematica website. July 31, 2023. Accessed August 4, 2023. https://www.mathematica.org/publications/medicaid-managed-care-enrollment-and-program-characteristics-2021

[11] Medicaid and CHIP Payment and Access Commission (MACPAC). Managed long-term services and supports. March 22, 2022. Accessed March 24, 2022. https://www.macpac.gov/subtopic/managed-long-term-services-and-supports/

[12] Enrollment report. Medicaid.gov. Accessed February 3, 2025. https://www.medicaid.gov/medicaid/managed-care/enrollment-report/index.html

[13] Freedman VA, Hu M, DeMatteis J, Kasper JD. Accounting for sample design in NHATS and NSOC analyses: frequently asked questions. Technical Paper #3. National Health & Aging Trends Study website. Accessed June 12, 2025. https://www.nhats.org/sites/default/files/2021-01/Accounting_for_the_NHATS_NSOC_Design_in_Analyses_FAQ_0.pdf

[14] Coe NB, Werner RM. Informal caregivers provide considerable front-line support in residential care facilities and nursing homes. Health Aff (Millwood). 2022;41(1):105-111. doi:10.1377/hlthaff.2021.01239 34982633 PMC8996285

[15] Kasper JD, Freedman VA, Spillman B. Classification of persons by dementia status in the national health and aging trends study. Technical Paper #5. National Health & Aging Trends Study website. July 2013. Accessed December 14, 2022. https://www.nhats.org/sites/default/files/inline-files/DementiaTechnicalPaperJuly_2_4_2013_10_23_15.pdf

[16] Freedman VA, Spillman BC, Kasper J. Hours of care in rounds 1 and 2 of the National Health and Aging Trends Study. Technical Paper #7. National Health & Aging Trends Study website. February 18, 2014. Accessed May 21, 2023. https://www.nhats.org/sites/default/files/2024-08/NHATSTechnicalPaper7_Aug2024.pdf

[17] Kaye N, Teshale S. Medicaid supports for family caregivers. National Academy for State Health Policy. October 2020. Accessed August 6, 2022. https://www.nashp.org/wp-content/uploads/2020/10/Medicaid-Supports-for-Family-Caregivers.pdf

[18] Shugrue N, Kellett K, Gruman C, . Progress and policy opportunities in family caregiver assessment: results from a national survey. J Appl Gerontol. 2019;38(9):1319-1341. doi:10.1177/0733464817733104 29165037

[19] Family Caregiver Alliance. Caregiver assessment: principles, guidelines and strategies for change. April 2006. Accessed August 13, 2023. https://www.caregiver.org/uploads/legacy/pdfs/v1_consensus.pdf

[20] Kasten J, Lewis E, Lelchook S, Feinberg L, Hado E. Recognition of family caregivers in managed long-term services and supports. AARP Public Policy Institute. April 16, 2020. Accessed August 1, 2025. https://www.aarp.org/pri/topics/ltss/family-caregiving/recognition-of-family-caregivers-in-managed-long-term-services-supports/

[21] Newcomer RJ, Kang T, Doty P. Allowing spouses to be paid personal care providers: spouse availability and effects on Medicaid-funded service use and expenditures. Gerontologist. 2012;52(4):517-530. doi:10.1093/geront/gnr102 22012960 PMC3530315

[22] Reinhard SC, Fox-Grage W, Feinberg LF. Family caregivers and managed long-term services and supports. AARP Public Policy Institute. Accessed July 20, 2022. https://www.aarp.org/content/dam/aarp/ppi/2016-08/AARP1080_FSandMLTSS_REPORT_WEB.pdf

[23] Schulz R, Eden J; National Academies of Sciences, Engineering, and Medicine, eds. Families Caring for an Aging America. The National Academies Press; 2016. doi:10.17226/23606 27905704

[24] Pluta-Ehlers L. Washington demonstrates cost savings and improved outcomes from supporting family caregivers. National Academy For State Health Policy (NASHP). October 8, 2021. Accessed February 10, 2025. https://nashp.org/washington-demonstrates-cost-savings-and-improved-outcomes-from-supporting-family-caregivers/

[25] Wolff JL, Cornman JC, Freedman VA. The number of family caregivers helping older US adults increased from 18 million to 24 million, 2011-22. Health Aff (Millwood). 2025;44(2):187-195. doi:10.1377/hlthaff.2024.00978 39899774 PMC11869104

[26] Leggett A, Koo HJ, Park B, Choi H. The changing tides of caregiving during the COVID-19 pandemic: How decreasing and increasing care provision relates to caregiver well-being. J Gerontol B Psychol Sci Soc Sci. 2022;77(suppl 1):S86-S97. doi:10.1093/geronb/gbac002 35032387 PMC9122649

[27] Truskinovsky Y, Wiemers EE. Paid care among older adults with long-term care needs declined in the first year of COVID-19 while families stepped in. Health Aff Sch. 2023;1(4):qxad040. doi:10.1093/haschl/qxad040 38756748 PMC10986229

[28] Kaye HS. Gradual rebalancing of Medicaid long-term services and supports saves money and serves more people, statistical model shows. Health Aff (Millwood). 2012;31(6):1195-1203. doi:10.1377/hlthaff.2011.1237 22665831

[29] Thompson F, Nadash P, Gusmano MK, Miller EA. Federalism and the growth of self-directed long-term services and supports. Public Policy Aging Rep. 2016;26(4):123-128. doi:10.1093/ppar/prw020

[30] Burns A, Mohamed M, Published MOW. Pandemic-era changes to Medicaid home- and community-based services (HCBS): a closer look at family caregiver policies. KFF. September 19, 2023. Accessed April 1, 2024. https://www.kff.org/medicaid/issue-brief/pandemic-era-changes-to-medicaid-home-and-community-based-services-hcbs-a-closer-look-at-family-caregiver-policies/

[31] Mellor J, Cunningham P, Britton E, Walker L. Use of home and community-based services after implementation of Medicaid managed long term services and supports in Virginia. J Aging Soc Policy. 2024;36(5):1026-1044. doi:10.1080/08959420.2023.2183678 36857515

[32] Amjad H, Wong SK, Roth DL, . Health services utilization in older adults with dementia receiving care coordination: the MIND at home trial. Health Serv Res. 2018;53(1):556-579. doi:10.1111/1475-6773.12647 28083879 PMC5785326

[33] Sciegaj M, Crisp S, DeLuca C, Mahoney KJ. Participant-directed services in managed long-term services and supports programs: a five state comparison. Office of the Assistant Secretary for Planning and Evaluation, U.S. Department of Health and Human Services. August 22, 2013. Accessed November 10, 2021. http://aspe.hhs.gov/reports/participant-directed-services-managed-long-term-services-supports-programs-five-state-comparison-1

[34] Sciegaj M, Crisp S, Edwards-Orr M, DeLuca C. Three emerging themes from implementing self-directed long-term service and support programs under managed care. Public Policy Aging Rep. 2016;26(4):134-137. doi:10.1093/ppar/prw019

[35] Kreider AR, Werner RM. The home care workforce has not kept pace with growth in home And community-based services. Health Aff (Millwood). 2023;42(5):650-657. doi:10.1377/hlthaff.2022.01351 37075251 PMC10278236

[36] Kasten J, Lipson D, Saucier P, Libersky J. Who enrolls in Medicaid managed care programs that cover long-term services and supports?: implications of enrollee diversity for a national cross-state evaluation. Medicaid.gov. June 2017. Accessed January 21, 2022. https://www.medicaid.gov/medicaid/downloads/1115-ib1-508-mltss-enrollment.pdf

[37] Anthony S, Traub A, Lewis S, et al. Strengthening Medicaid Long-Term Services and Supports in an Evolving Policy Environment: A Toolkit for States. Manatt Health Strategies and Center for Health Care Strategies, Inc; 2019:82.

[38] Yocom CL. Medicaid long-term services and supports: access and quality problems in managed care demand improved oversight. United States Government Accountability Office. November 2020. Accessed December 20, 2021. https://www.gao.gov/assets/gao-21-49.pdf

[39] Gonzalez L, Polivka-West L, Polivka L. Failures of regulation and policy in Medicaid-managed long-term care and Medicare Advantage. Public Policy Aging Rep. 2021;31(2):57-61. doi:10.1093/ppar/prab002

[40] Bowers A, Owen R, Heller T. Care coordination experiences of people with disabilities enrolled in Medicaid managed care. Disabil Rehabil. 2017;39(21):2207-2214. doi:10.1080/09638288.2016.1219773 27548093

[41] Call KT, Davern ME, Klerman JA, Lynch V. Comparing errors in Medicaid reporting across surveys: evidence to date. Health Serv Res. 2013;48(2 Pt 1):652-664. doi:10.1111/j.1475-6773.2012.01446.x 22816493 PMC3626347

