# Supplementary material for: Managed Long-Term Services and Supports and Caregiving Among Dually Enrolled Older Adults
Source: JAMA Netw Open. 2025 Aug 21;8(8):e2528006. doi: 10.1001/jamanetworkopen.2025.28006 (PMC12371512; doi:10.1001/jamanetworkopen.2025.28006)
Supplement: Supplement 1. — eTable 1. Percentage of Older Dual-Enrollees Receiving Assistance in the Community By Residence in Managed Long-term Services and Support (MLTSS) Program Presence and Survey Round eTable 2. Sociodemographic, Health, and Function Characteristics Among Older Dual-Enrollees Receiving Assistance in the Community by Survey Round eTable 3. Caregiving Characteristics Among Older Dual-Enrollees Receiving Assistance in the Community by Survey Round [file jamanetwopen-e2528006-s001.pdf]

Supplemental Online Content

Jopson AD, Fabius CD, Wolff JL. Managed Long-Term Services and Supports and Caregiving Among Dually-Enrolled Older Adults. *JAMA Netw Open*. 2025;8(8):e2528006. doi: 10.1001/jamanetworkopen.2025.28006

eTable 1. Percentage of Older Dual-Enrollees Receiving Assistance in the Community By Residence in Managed Long-term Services and Support (MLTSS) Program Presence and Survey Round
eTable 2. Sociodemographic, Health, and Function Characteristics Among Older Dual-Enrollees Receiving Assistance in the Community by Survey Round
eTable 3. Caregiving Characteristics Among Older Dual-Enrollees Receiving Assistance in the Community by Survey Round

This supplemental material has been provided by the authors to give readers additional information about their work.

eTable1. Weighted Percentages of Older Dual-Enrollees Receiving Assistance By Residence in Areas with MLTSS Program Presence By Survey Round (N=2,549)

Year	2012	2013	2014	2015	2016	2017	2018	2019	2020	2021	2022
NHATS Survey Round	2	3	4	5	6	7	8	9	10	11	12
Unweighted number of observations	331	283	245	375	349	317	316	329	287	207	328
Weighted estimates (000s)	913	1,017	899	944	971	1,010	1,118	1,363	1,173	1,027	1,146
**MLTSS Program Presence**											
No MLTSS Program Presence	60.6	59.7	48.6	36.8	30.3	31.4	33.6	34.1	32.3	29.0	28.6
Weighted estimates (000s)	554	607	436	347	294	317	376	465	379	297	328
MLTSS Program Presence	39.4	40.3	51.4	63.2	69.7	68.6	66.4	65.9	67.7	71.0	71.4
Weighted estimates (000s)	360	410	462	597	677	693	742	899	794	729	818
**MLTSS Enrollment Type**											
No MLTSS	60.6	59.7	48.6	36.8	30.3	31.4	33.6	34.1	32.3	29.0	28.6
Voluntary	20.6	14.5	14.7	11.2	16.1	15.0	16.8	9.6	11.0	9.5	11.6
Mandatory[a]	18.8	25.8	36.7	52.0	53.6	53.6	49.6	56.3	56.7	61.5	59.8
**MLTSS Program Adoption During Study Period[b]**											
No MLTSS	30.8	30.7	29.3	29.7	25.2	27.2	29.6	32.5	32.2	28.8	28.0
Added MLTSS	29.8	29.0	33.0	29.1	37.2	33.7	33.1	28.1	28.5	28.7	28.7
Continuous MLTSS	39.4	40.3	37.6	41.1	37.7	39.1	37.3	39.4	39.3	42.5	43.3

[a] Areas with voluntary and mandatory programs were classified as mandatory.
[b] Multiple states added and expanded MLTSS programs during the observation period. We classified counties into three categories based on whether they did or did not have MLTSS for each year. Counties with "Continuous MLTSS" had MLTSS programs present throughout the observation period. Counties that "Added MLTSS" added MLTSS during the study period. Counties with "No MLTSS" did not have any MLTSS programs present throughout the study period.

eTable 2. Sociodemographic, Health, and Function Characteristics Among Older Dual-Enrollees Receiving Assistance by Survey Round (N=2,549)

Year	2012	2013	2014	2015	2016	2017	2018	2019	2020	2021	2022	All Years
NHATS Survey Round	2	3	4	5	6	7	8	9	10	11	12	All Rounds
Unweighted observations	270	222	171	296	268	235	235	236	202	151	263	2,549
Weighted estimates (000s)	913	1,016	898	944	971	1,010	1,117	1,363	1,172	1,026	1,146	11,581
**Age, mean (SD)**	81.4 (9.0)	80.1 (8.2)	80.4 (8.1)	79.9 (10.1)	81.4 (9.8)	80.7 (9.5)	79.5 (8.5)	79.0 (7.8)	80.6 (7.6)	80.2 (6.7)	80.4 (8.2)	80.3 (8.5)
**Sex (%)**												
Male	61 (27.7)	44 (20.8)	40 (25.8)	79 (31.1)	67 (29.3)	52 (23.1)	59 (32.6)	64 (36.6)	56 (35.5)	46 (41.6)	58 (21.6)	626 (29.9)
Female	209 (72.3)	178 (79.2)	131 (74.2)	217 (68.9)	201 (70.7)	183 (76.9)	176 (67.4)	172 (63.4)	146 (64.5)	105 (58.4)	205 (78.4)	1,923 (70.1)
**Race/Ethnicity (%)**												
Non-Hispanic White	74 (40.8)	68 (45.7)	44 (35.8)	70 (35.3)	73 (37.4)	59 (33.8)	56 (35.5)	63 (35.4)	57 (34.5)	39 (35.2)	48 (36.5)	651 (36.8)
Non-Hispanic Black	123 (22.1)	96 (19.9)	75 (19.7)	139 (22.1)	121 (21.9)	111 (21.3)	123 (25.0)	103 (18.4)	89 (20.5)	73 (20.7)	109 (21.9)	1,162 (21.2)
Hispanic and other	73 (37.1)	58 (34.4)	52 (44.5)	87 (42.6)	74 (40.8)	65 (44.9)	56 (39.5)	70 (46.3)	56 (45.0)	39 (44.1)	106 (41.6)	736 (42.0)
**Living Arrangement (%)**												
Lives alone	74 (29.6)	49 (22.9)	40 (22.0)	72 (24.7)	66 (23.5)	58 (21.3)	53 (22.9)	51 (23.9)	60 (31.2)	53 (40.9)	70 (28.1)	646 (26.5)
Lives with others	196 (70.4)	173 (77.1)	131 (78.0)	224 (75.3)	202 (76.5)	177 (78.7)	182 (77.1)	185 (76.1)	142 (68.8)	98 (59.1)	193 (71.9)	1,903 (73.5)
**Dementia status (%)**												

No Dementia	158 (62.4)	133 (63.2)	100 (59.5)	173 (60.5)	162 (62.1)	151 (67.0%)	155 (69.4%)	161 (78.5)	125 (65.6)	103 (69.8)	162 (62.3)	1,583 (66.0)
Dementia	112 (37.6)	89 (36.8)	71 (40.5)	123 (39.5)	106 (37.9)	84 (33.0)	80 (30.6)	75 (21.5)	77 (34.4)	48 (30.2)	101 (37.7)	966 (34.0)
**Chronic Conditions, (%)**												
0-2 conditions	86 (30.1)	52 (21.6)	39 (22.8)	74 (23.2)	64 (22.9)	46 (15.3)	39 (13.4)	36 (12.2)	33 (15.0)	21 (12.2)	72 (31.2)	562 (19.6)
3-4 conditions	137 (51.1)	131 (60.7)	96 (56.6)	177 (60.8)	158 (57.3)	147 (64.0)	140 (58.1)	144 (62.1)	112 (58.0)	87 (54.0)	131 (51.2)	1,460 (57.7)
5+ conditions	47 (18.8)	39 (17.7)	36 (20.7)	45 (16.0)	46 (19.8)	42 (20.7)	56 (28.4)	56 (25.6)	57 (27.1)	43 (33.9)	60 (17.7)	527 (22.7)
**Number of self-care or mobility activities receiving assistance, mean (SD)**	2.7 (2.3)	2.8 (2.1)	3.0 (1.9)	2.9 (2.5)	2.9 (2.4)	2.9 (2.1)	2.9 (2.1)	2.5 (1.7)	2.7 (1.8)	2.7 (1.6)	2.9 (2.0)	2.8 (2.0)

eTable 3. Caregiving Characteristics Among Older Dual-Enrollees Receiving Assistance and Living in the Community by Survey Round (N=2,549)

Year	2012	2013	2014	2015	2016	2017	2018	2019	2020	2021	2022	All Years
NHATS Survey Round	2	3	4	5	6	7	8	9	10	11	12	All Rounds
Unweighted observations	270	222	171	296	268	235	235	236	202	151	263	2,549
Weighted estimates (000s)	913	1,016	898	944	971	1,010	1,117	1,363	1,172	1,026	1,146	11,581
**Receipt of paid help (%)**												
Did not receive paid help	138 (52.2)	152 (73.6)	112 (66.2)	135 (43.1)	174 (64.8)	98 (44.4)	100 (41.8)	81 (35.0)	85 (45.4)	62 (45.7)	110 (47.4)	1,247 (50.0)
Received paid help	132 (47.8)	70 (26.4)	59 (33.8)	161 (56.9)	94 (35.2)	137 (55.6)	135 (58.2)	155 (65.0)	117 (54.6)	89 (54.3)	153 (52.6)	1,302 (50.0)
**Total number of caregivers, mean (SD)**	2.9 (1.9)	2.7 (1.6)	2.9 (1.5)	3.0 (2.2)	3.0 (2.0)	2.8 (1.6)	3.0 (1.5)	2.9 (1.5)	2.7 (1.4)	2.6 (1.4)	2.7 (1.8)	2.8 (1.7)
**Paid caregivers, mean (SD)**	0.7 (1.3)	0.3 (0.6)	0.4 (0.8)	0.8 (1.3)	0.4 (0.8)	0.8 (1.0)	0.8 (1.0)	0.8 (0.8)	0.8 (0.9)	0.7 (0.8)	0.7 (1.0)	0.7 (1.0)
**Unpaid caregivers, mean (SD)**	2.2 (1.8)	2.4 (1.6)	2.5 (1.5)	2.1 (2.0)	2.6 (2.0)	2.1 (1.7)	2.2 (1.6)	2.0 (1.5)	2.0 (1.4)	1.9 (1.3)	2.0 (1.9)	2.2 (1.7)
**Care Hours Received Per Week**												

**All caregivers, mean (SD)**	67.1 (93.0)	70.0 (79.6)	79.9 (82.0)	70.8 (109.5)	65.4 (86.8)	66.6 (75.3)	68.0 (82.1)	59.5 (63.3)	58.1 (66.5)	49.0 (50.5)	71.8 (99.4)	65.6 (80.9)
**Paid family caregivers, mean (SD)**	3.2 (18.0)	8.4 (32.9)	9.0 (32.6)	7.2 (37.3)	8.5 (33.8)	6.8 (29.3)	8.6 (32.9)	7.5 (26.0)	6.7 (23.3)	4.5 (13.0)	17.6 (51.4)	8.1 (31.8)
**Unpaid family caregivers, mean (SD)**	49.6 (89.5)	45.7 (73.9)	47.6 (74.3)	47.6 (102.3)	38.2 (77.9)	47.1 (73.8)	43.4 (78.9)	37.6 (55.2)	37.0 (60.2)	28.1 (46.5)	43.4 (84.5)	42.0 (74.0)
**Paid other caregivers, mean (SD)**	12.8 (41.9)	2.2 (16.8)	6.0 (26.5)	13.9 (39.6)	4.2 (20.6)	12.0 (28.6)	15.5 (37.6)	12.4 (27.9)	12.6 (27.3)	13.6 (28.1)	8.6 (25.9)	10.5 (30.4)
**Unpaid other caregivers, mean (SD)**	1.5 (8.8)	13.7 (33.5)	17.4 (39.3)	2.0 (16.2)	14.5 (41.4)	0.7 (5.3)	0.5 (3.5)	1.9 (11.9)	1.8 (11.4)	2.8 (9.8)	2.3 (13.4)	5.1 (22.2)
