# Supplementary material for: Managed Long-Term Services and Supports and Caregiving Among Dually Enrolled Older Adults
Source: JAMA Netw Open. 2025 Aug 21;8(8):e2528006. doi: 10.1001/jamanetworkopen.2025.28006 (PMC12371512; doi:10.1001/jamanetworkopen.2025.28006)
Supplement: Supplement 2. — Data Sharing Statement [file jamanetwopen-e2528006-s002.pdf]

Data Sharing Statement

Jopson. Managed Long-Term Services and Supports and Caregiving Among Dually-Enrolled Older Adults. *JAMA Netw Open*. Published August 21, 2025.
doi:10.1001/jamanetworkopen.2025.28006

Data
Data available: No

Additional Information
Explanation for why data not available: It is already publicly available.
